# The Acute Effects of Exercise Intensity on Inhibitory Cognitive Control in Adolescents

**DOI:** 10.3389/fpsyg.2017.00921

**Published:** 2017-05-31

**Authors:** Fernando Peruyero, Julio Zapata, Diego Pastor, Eduardo Cervelló

**Affiliations:** Sport Research Center, Miguel Hernandez University of ElcheElche, Spain

**Keywords:** adolescence, physical education, cognition, Stroop test, intensity

## Abstract

Adolescence is an important stage for brain maturation. There are many studies of exercise-cognition relations, but there is still a lack of knowledge about the impact of combining different intensities of exercise on adolescents’ cognitive responses. The main objective of this study was to analyze the effect of three physical education sessions (based on Zumba dance) of different intensities (no exercise, predominantly light intensity, and predominantly vigorous intensity) on the inhibition response (measured with the Stroop test) in adolescents. Forty-four adolescent students (age 16.39 ± 0.68) completed a Stroop test before and after the three different physical education sessions. The results show than the predominantly vigorous session represented the strongest stimulus to increase cognitive inhibitory control. This means that the cognitive effect of exercise can be conditioned by exercise intensity and implies the need to control exercise intensity in physical educational programs for adolescents.

## Introduction

There is currently a consensus in the scientific community about the positive effects of physical exercise on cognition (Chang et al., 2012; Verburgh et al., 2014). However, few studies focus on the acute effects of physical exercise and its impact on higher cognitive functions (Chu et al., 2015; Davranche et al., 2015; Howie et al., 2015; Piepmeier et al., 2015; Pontifex et al., 2015). Indeed, more studies are needed to understand the relations between intensities, durations and type of exercise and executive control in adolescents (Chang et al., 2015; Diamond, 2015; McMorris and Hale, 2015; Domazet et al., 2016; Harveson et al., 2016). The study of the dose-response relations in exercise is a current challenge in the science of physical activity (Curlik and Shors, 2013).

Executive functions can be conceptualized as a set of processes that enable one to plan, coordinate, sequence, and monitor cognitive operations (Stuss, 1992; Boucard et al., 2012). Diamond (2013, 2015) distinguished three latent variables related to executive control: *inhibition*, which includes selective attention, resisting distractions, and staying focused; *working memory;* and *cognitive flexibility.* Inhibition is related to the capacity to suppress irrelevant information and response tendency.

Recent studies highlight the significance of inhibitory control in adolescents’ academic outcomes, particularly in mathematics (Cragg and Gilmore, 2014; Domazet et al., 2016) and efficiency in managing the oral language system (Berninger et al., 2016).

Domazet et al.’s (2016) studied habitual physical exercise, objectively measured, in relation to inhibitory cognitive function and found no significant relations between the amount of exercise and inhibitory control but there was a relationship with academic outcomes in math. The author stressed the need to study exercise frequency and intensity in relation to inhibitory function, in accordance with the review of Diamond (2015), who detected the general limitation of these studies of exercise and executive function, which do not usually study the relation between different kinds of exercise and cognition.

In fact, there is evidence that only one session of aerobic physical activity improves cognitive performance in spatial attention tasks (Tsai et al., 2014; Llorens et al., 2015), learning (Winter et al., 2007), episodic memory (Griffin et al., 2011; Weinberg et al., 2014), and executive and inhibitory function (Sibley et al., 2006; Ferris et al., 2007; van Uffelen et al., 2008; Baker et al., 2010; Liu-Ambrose et al., 2010; Griffin et al., 2011; Chang et al., 2015; Weng et al., 2015; Sandroff et al., 2016). However, there are very few studies with adolescents (Cooper et al., 2016a,b) and preadolescents (Niemann et al., 2013).

Time is also linked to acute exercise effect. Chang et al. (2015) proposed the optimum duration of an acute bout of exercise to improve cognitive performance. No less than 20 min and no more than 40 are necessary to observe significant improvements.

As well as time, intensity can modulate the effect of exercise on cognitive function. Moderate intensity seems to be the most efficient to improve cognition (Chmura et al., 1994; Arent and Landers, 2003; Chang et al., 2015) but some articles failed to find differences in inhibitory control between an acute aerobic task of moderate intensity and a passive task (Weng et al., 2015). In contrast, recent studies have shown that vigorous intensities and different combinations of intensities can improve inhibitory control tasks (Sandroff et al., 2016; Tsukamoto et al., 2016). Furthermore, a linear increase of intensity in an acute bout of exercise did not imply a decline in inhibitory control (Schmit et al., 2015).

There are few studies on the relation between exercise intensity and inhibitory control in adolescents. Some studies have analyzed the acute improvement of some higher executive functions with physical exercise sessions of moderate intensities (Verburgh et al., 2014; Cooper et al., 2016b). However, there are even fewer articles about inhibitory control (Cooper et al., 2016a). In contrast, Soga’s group found cognitive improvements in adolescents during moderate exercise, but the improvements were not maintained after the end of the exercise session (Soga et al., 2015).

The previous contradictory results and the challenge to improve our knowledge of dose-response relations between intensity and inhibitory control in adolescents have led us to propose this paper, with the aim of studying the relations between exercise intensity and inhibitory control cognition in three acute sessions of physical education, with adolescents in a real school environment.

We hypothesize that students in the physical education class with predominately vigorous intensity will show greater inhibitory control than a control class with predominately light intensity.

## Materials and Methods

### Participants’ Demographics

Forty-four students (23 boys and 21 girls) from four classes (age 16.39 ± 0.68 years) participated in this study. All the students from all four classes were informed about the project. They received a written informed consent to be completed by their parents and all of them knew they would participate in the theoretical session and the aerobic-zumba session as part of their normal classes.

Participants did not undergo any risk test, but they usually participate in high-intensity activities in schoolar physical exercise classrooms. Moreover, in the written informed consent intensity and its risk were explained to the participants and their legal tutors. On the other hand, the spanish public education system requires a medical inform for students that have particular health risk related to physical activity practice. These students did not participated in the research.

Only the students who brought the written informed consent were measured (61 in total) but all of them participated in the sessions. Only the students who participated in all three sessions were included in the study, so, finally we only present the results of 44 students. There were no reward because they participated as part of their normal educational process.

The schools are located in a large city of Spain. All of them are public centers with students of middle economic class. Of course, not all the students are from the same socio-economic class, but all of them are from the city center and have a similar economic status (in Spanish public schools, students are admitted by proximity to their home).

### Material and Measures

#### Cognitive Inhibition (*Stroop Test*)

The Spanish adaptation of Golden’s test was used in this study (Golden, 1994). It is one of the most commonly used neuropsychological instruments to measure multiple cognitive processes, including information-processing speed, executive control, selective attention, and the ability to inhibit habitual responses (Pachana et al., 2004; Chang et al., 2015; Tsukamoto et al., 2016). We used the pencil-and-paper version of the Stroop task. In the “word” or “congruent” condition, the words (e.g., the Spanish word for RED, BLUE, and GREEN) were written in black ink. Performance of this task is highly dependent on information-processing speed. In the “color” condition, sequences of XXX were written in red, blue, or green ink (neutral condition). In the “color-word” or “incongruent” condition, the three color words systematically differed from the word’s ink color (e.g., the Spanish word for RED written in the color blue). Participants were requested to read the words in the word condition and to name the ink color in the other conditions aloud as quickly as possible during 45 s, and the number of correct responses was recorded. The dependent measures had three levels: (1) Neutral condition, (2) Congruent condition, and (3) Incongruent condition.

The Spanish version of this Stroop test has been recently validated (Rodríguez Barreto et al., 2016). This test measures the correct responses in 45 s, and there is no limit to the number of possible responses. This version of the Stroop task does not allow us to know the reaction time after presenting the stimulus. Studies of the reliability and validity of the instrument show it is good tool to measure inhibitory cognition with three increasingly complex tasks (Congruent, Neutral, and Incongruent), showing a high intra-class correlation index for different temporal measures (>0.90) (Rodríguez Barreto et al., 2016).

### Procedure

The study was approved by the university ethical committee and was implemented in accordance with the Declaration of Helsinki. Written informed consent was obtained from the students and their parents prior to the study. All the subjects participated in four sessions (one pre-experimental session and three experimental sessions).

#### Pre-experimental Session

The first session was a training session in which the participants completed their sociodemographic data and performed a training session in the Stroop test to prevent the learning effect in the rest of the sessions. Each participant completed at least two blocks of 80 trials. When they performed two consecutive blocks with less than 5% intra-block variability in the second block with regard to the previous block, the training was concluded (3.48 ± 1.09 trials). By means of this procedure, we respected individual variability in the learning process. Previous studies have used the same intra-variability percentage as a measure of learning stability in inhibition cognitive tests (Schmit et al., 2015).

#### Experimental Sessions

The other three sessions consisted of: (a) a 20-min session of physical education with only theoretical contents [non-exercise (NE)]; (b) a 5-min warm-up session, 20 min of light-to-moderate intensity exercise (LM) and a 5-min cool-down; and (c) a session like “b” but with moderate-to-vigorous intensity exercise (MV). The physical activity sessions were “aerobic-zumba,” in which participants replicated the instructor’s movements, with different intensities. The students have in their academic contents one dance didactic unity, this year the academic professors selected Zumba for this didactic unity. So we use the same academic objectives that thay also had programmed. Zumba was not a researcher objective, not selected by researchers, it was programmed in their studies and we adapted to them.

All three sessions included a pre- and post-session measure of the Stroop test, just before warm up, and just after cool down, or 5 min previous and posterior the NE session. All sessions were led by team researchers.

The NE session consisted of 15 slides of health tips related to exercise, nutrition, sedentary lifestyle, and hygiene. Sessions (b) and (c) were recorded with accelerometers to subsequently confirm intensity.

All subjects participated in all three sessions but they were divided into three groups to counterbalance session, avoiding the possible influence of session order (Thomas et al., 2015).

### Duration and Intensity Control

A pilot study was carried out prior to the study to set the intensities of the exercises and the recovery times to ensure that the goals of the sessions were met. Easy aerobic exercises were selected for the sessions, and participants were instructed to imitate the instructor. All participants used an accelerometer during the sessions to ensure that all of them achieved the correct intensity.

ActiGraph accelerometers GT3X were used in this study. Participants were instructed in their correct use at the beginning of each session (Santos-Lozano et al., 2013). Epoch length of 1 s was used, as some authors recommend (Sallis et al., 1999; Dorsey et al., 2009; Torres-Luque et al., 2014). Accelerometers were programmed to record 30 min, and the instructor was trained to start the last 2 min specific warm-up right at the beginning of the accelerometer’s register (accelerometers were programmed by computer before the session, and the instructor’s watch was synchronized). After the session, the first 24 min were analyzed, which included the 2-min warm-up, the 20-min session, and the 2-min cool-down.

At least 20 min’ wear-time of activity were required to include the subject in the study. Epochs smaller than 15 counts and inactivity periods longer than 1 min were ignored. ActiLife 6 (Actigraph, ActiLife version 6.11.5) software was used to analyze the data, and physical activity intensity (light, moderate, and vigorous) was defined using Troiano et al.’s (2008) thresholds. These authors’ method was selected because it is clear about the cut-points for each age category (Treuth et al., 2004; Kremer et al., 2012; Torres-Luque et al., 2014). Specific criteria for each age can be found in^1^.

**FIGURE 1:**
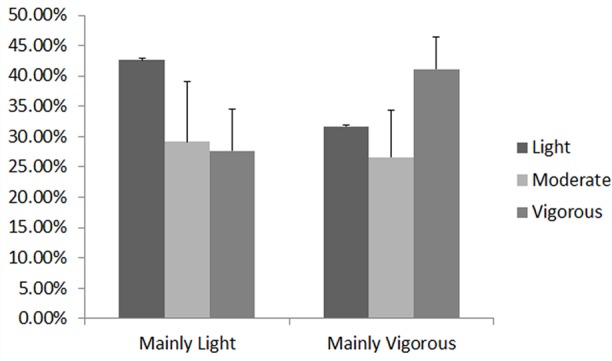
Percentages of time at different intensities in sessions mainly light and mainly vigorous.

**Figure 1** presents the percentages of time at different intensities in each session. As can be seen, both sessions (sessions two and three) had the same percentage of moderate intensity, and different percentages of light and vigorous time (called “mainly light” and “mainly vigorous,” respectively). The goal was to determine whether different times of vigorous or light intensities affect cognition differently, maintaining the time of moderate intensity, which the bibliography presents as the most efficient for cognitive performance (Chang et al., 2015).

### Data Analysis

Descriptive data are presented as mean ± SE. A 2 × 3 × 3 (Time: pre–post × Treatment intensity: no exercise, mainly light and mainly vigorous × Stroop test condition: congruent, neutral, and incongruent) repeated-measures ANOVA was calculated to analyze differences in accuracy in the Stroop test. Paired *post hoc t*-tests with Bonferroni adjustments for multiple comparisons were performed to follow up significant effects. Effect sizes are expressed as partial eta-squared ($\eta_{p}^{2}$), and an alpha of 0.05 was used for the significance level. Paired *t*-tests were performed to clarify the pre–post effect of the different intervention groups. Statistical data are shown in **Table 2**, which presents the *t-*value, the *p*-value, and the effect size with the confidence interval.

## Results

### Stroop Test Performance for Intensity Conditions

Descriptive data for measures of response accuracy in the Stroop test based on treatment are presented in **Table 1**.

**Table 1:** Mean and (SE) values for Stroop test Performance in all sessions (*N* = 44).

	Pre			Post		
Variables	No exercise	Mainly light	Mainly vigorous	No exercise	Mainly light	Mainly vigorous
Congruent condition	57.93 (1.51)	58.72 (1.75)	62.18 (1.61)	61.09 (1.51)	66.86^∗^ (1.72)	76.95^∗^ (0.72)
Neutral condition	55.68 (1.04)	50.22 (1.35)	55.18 (1.44)	58.45 (1.51)	55.33^∗^ (1.37)	65.29^∗^ (1.70)
Incongruent condition	56.06 (1.00)	51.00 (1.34)	53.50 (1.46)	60.45^∗^ (1.32)	62.15^∗^ (1.54)	71.00^∗^ (1.50)

*Data is Stroop accuracy, measured as successes less mistakes in 45 s. ^∗^*p* < 0.05 in the pre–post analysis.*

The 2 × 3 × 3 ANOVA indicated a statistically non-significant Time × Stroop Condition × Treatment Intensity interaction [*F*(4,40) = 1.68, *p* > 0.173, $\eta_{p}^{2}$ = 0.14]. On another hand, results indicated a significant Stroop Condition × Treatment Intensity interaction [*F*(4,40) = 6.90, *p* < 0.001, $\eta_{p}^{2}$ = 0.40] in response accuracy, and differences in accuracy related to Treatment Intensity [*F*(2,44) = 74.82, *p* < 0.001, $\eta_{p}^{2}$ = 0.78], Stroop Condition [*F*(2,44) = 53.85, *p* < 0.001, $\eta_{p}^{2}$ = 0.71], and a large effect of Time [*F*(1,43) = 114.72, *p* < 0.001, $\eta_{p}^{2}$ = 0.72], indicating that accuracy increased when comparing the pre and post situations.

**Table 2** shows that there were significant pre–post differences according to exercise intensity, and these differences were higher with high intensity (effect size). In the NE session, there were differences only in the incongruent condition.

**Table 2:** *p*-value, Cohen’s effect size (d) and Confidence Interval (CI) lower and upper, for Stroop test Performance (*N* = 44).

Non exercise session	*t*	*p*-Value	Effect size	CI lower	CI upper
Congruent condition	–1.894	0.065	0.31	–0.11	0.73
Neutral condition	–1.541	0.131	0.32	–0.11	0.74
Incongruent condition	–3.092	0.003	0.56	0.13	0.98
**Light to moderate session**					
Congruent condition	–4.803	0.001	0.70	0.27	1.13
Neutral condition	–4.183	0.001	0.57	0.13	0.99
Incongruent condition	–6.941	0.001	1.16	0.70	1.60
**Moderate to vigorous session**					
Congruent condition	–8.923	0.001	1.78	1.27	2.26
Neutral condition	–5.325	0.001	0.95	0.50	1.38
Incongruent condition	–6.328	0.001	1.78	1.27	2.26

Multiple follow-up comparisons showed that, when intensity increased, the accuracy of responses also increased, but only moderate-to-vigorous intensities showed significant differences. Accuracy was significantly higher in the moderate-to-vigorous session when compared with the no-exercise condition (*p* < 0.001) and with the session of light-to-moderate intensity (*p* < 0.001). When comparing the NE condition with the light-to-moderate session, the results were better in the light-to-moderate session but the differences were non-significant (*p* > 0.285).

When comparing the effect of the Stroop condition on accuracy, only the congruent condition obtained better accuracy scores than the neutral condition (*p* < 0.001) and than the incongruent condition (*p* < 0.001).

## Discussion

The main objective of this study is to analyze the effect of three different intensities of physical education sessions, with same duration, on an inhibitory control task performed by adolescent students in a real environment.

The main contribution of this work is to prove, in a real ecological environment with adolescent population, the relevance of exercise intensity for inhibitory control when session time is maintained within the recommended values. Vigorous activity was shown to have a higher impact than light activity on cognitive function (Chang et al., 2015).

In this situation, the moderate-to-vigorous session produced significant improvements in cognitive inhibition compared both with the light-to-moderate intensity and NE sessions.

The effect of exercise in the congruent condition is noteworthy, with a high effect size. These results are in agreement with the study of Sandroff et al. (2016), who also found improvements of inhibitory control in congruent conditions regardless of exercise intensity. This is not surprising because less inhibitory control is needed to perform the congruent task than for the incongruent condition.

Results also reveal that all exercise sessions improved cognitive response. The results show a positive effect of exercise on inhibitory cognitive function when pre- and post-intervention are compared (Time), either in light-to-moderate or in moderate-to-vigorous, with high effect sizes. We also found that cognitive inhibition improved in the incongruent condition of the NE situation. So, other factors instead of physical exercise must be playing a significant role in this situation. In fact, Koutsandreou et al. (2016) showed that low intensity exercises with high cognitive challenge are related to motor control. That means that cognitive interventions or motor learning intervention can also have a large impact on cognitive responses.

Our results have also shown stronger improvements with higher intensities. Our results are in agreement with a recent study of Tsukamoto’s group (Tsukamoto et al., 2016). This study involve 12 healthy male subjects (22.9 ± 0.4 years) that participated in two different acute exercise protocols (a continuous exercise protocol of 40 min at 60% VO_2_max peak versus a high interval intensity protocol with four repetitions of 4 min at 90% VO_2_max peak with 3 min of active recovery at 60% VO_2_max). Both intensities improve inhibitory control, but the high interval session (combination of intensities) showed better results in the next 30 min. As our results, this study emphasizes the relevance of high intensities to better improvements in cognitive responses. Similar with our study, HIIT is a combination of intensities predominantly vigorous, showing that, independently of exercise modalities, the key to improve inhibitory control is the control of exercise intensity.

In accordance with these results, Schmit et al. (2015) also found that high intensity exercise improved cognitive performance, although intensity can produce poor cognitive results if it reaches the point of exhaustion. Consistent with this author, as intensity did not reach the point of exhaustion, our participants did not show any cognitive impairment despite the intensity. According to these authors, intensity seems to be more or less adaptive in relation to other factors, such as duration and subject fitness, always avoiding exhaustion (Schmit et al., 2015). In fact, some previous works have considered the relevance of fitness as a variable in the acute exercise effect (Chang et al., 2012). In our study, we tried to control fatigue by limiting exercise duration and combining intensities, which is also more ecological within a habitual physical education class.

Future research should study in detail combinations of intensities, beyond only comparing different intensities, because intensity combination reflects a more ecological situation of real exercise practice. This information can help to more accurately adjust exercise training programs, especially programs oriented toward cognitive inhibition in particular, or toward more global cognitive function.

One of the aspects of this research that should be studied in the future is the effect of exercise on inhibitory control in adolescents. This developmental stage is particularly important because the brain structures are maturing, especially regarding executive function and social cognition (Blakemore and Choudhury, 2006). It is necessary to study the acute and long-term effects of exercise during this important stage. Indeed, some studies refer to the acute effect of exercise on neurotrophic factors (Phillips et al., 2014; Soga et al., 2015), such as brain derived neurotrophic factor (BDNF) (Schmolesky et al., 2013; Szuhany et al., 2015). We have shown that the acute effect of exercise on neurotrophic release in inhibitory control is also mediated by intensity (Heyman et al., 2012; Schmolesky et al., 2013), and the importance of these factors in cognitive development (Lee et al., 2014).

The relevant contribution of the study (the combination of intensities in ecological physical exercise classes) is also a limitation of the study. This limitation is inherent to every ecological study and is related to the attribution of cognitive changes to a specific intensity. In both exercise protocols, the moderate intensity was stable, and only light and vigorous intensity changed. We cannot determine whether cognitive improvement is produced in both sessions due to the presence of vigorous intensity (although it is present in different amounts). It is known that vigorous-intensity exercise improves cognitive function (Browne et al., 2016). The warm up and cool down extra time in the exercise session, maybe can be a limitation respect the NE session, as the experiment only compare the 20 min intervention. The lack of anthropometric information is another aspect that must be studied in the future in relation with recent study who show the relation between obesity in adolescents and cognitive responses (Sweat et al., 2017).

The absence of effort response, subjective like RPE (Borg and Noble, 1974) or objective (like Heart Rate) is another limitation of the study that must be analyzed in future research, to find the relation between individual effort and cognitive improvements.

Nevertheless, our study shows greater improvements with higher amounts of vigorous intensity. These relations have been studied previously (Chang and Etnier, 2009) but is still necessary to understand the dose-response relations between exercise intensity and inhibitory cognition and, more interestingly, in ecological environments.

The pencil-and-paper test is another limitation because it provides the correct responses of the test but we cannot determine the reaction time or the relation of correct/incorrect responses, as are usually studied with software tests.

## Conclusion

This study analyzes the combination of different intensities of acute exercise in an environment of a physical education class, in relation to adolescents’ cognitive function. We show that intensity is related to improvement in inhibitory control, and this improvement depends on exercise intensity. That is, vigorous intensity seems to be better to increase cognition. Future research should continue to study this hypothesis to improve the school programs of physical activity for adolescents.

## Author Contributions

FP and JZ participated in design, acquisition and analysis of the study. EC and DP participated in design, drafting and critical revision of the manuscript.

## Conflict of Interest Statement

The authors declare that the research was conducted in the absence of any commercial or financial relationships that could be construed as a potential conflict of interest. The reviewer MKL and handling Editor declared their shared affiliation, and the handling Editor states that the process nevertheless met the standards of a fair and objective review.

## Footnotes

1 http://epi.grants.cancer.gov/nhanes_pam/

## References

[B1] Arent S. M. Landers D. M. (2003). Arousal, anxiety, and performance: a reexamination of the Inverted-U hypothesis. *Res. Q. Exerc. Sport* 74 436–444. 10.1080/02701367.2003.10609113 14768844

[B2] Baker L. D. Frank L. L. Foster-Schubert K. Green P. S. Wilkinson C. W. McTiernan A. (2010). Effects of aerobic exercise on mild cognitive impairment: a controlled trial. *Arch. Neurol.* 67 71–79. 10.1001/archneurol.2009.307 20065132 PMC3056436

[B3] Berninger V. Abbott R. Cook C. R. Nagy W. (2016). Relationships of attention and executive functions to oral language, reading, and writing skills and systems in middle childhood and early adolescence. *J. Learn. Disabil.* 10.1177/0022219415617167 [Epub ahead of print]. PMC4938801 26746315

[B4] Blakemore S. J. Choudhury S. (2006). Development of the adolescent brain: implications for executive function and social cognition. *J. Child Psychol. Psychiatry* 47 296–312. 10.1111/j.1469-7610.2006.01611.x 16492261

[B5] Borg G. A. Noble B. J. (1974). Perceived exertion. *Exerc. Sport Sci. Rev.* 2 131–154. 10.1249/00003677-197400020-00006 4466663

[B6] Boucard G. K. Albinet C. T. Bugaiska A. Bouquet C. A. Clarys D. Audiffren M. (2012). Impact of physical activity on executive functions in aging: a selective effect on inhibition among old adults. *J. Sport Exerc. Psychol.* 34 808–827. 10.1123/jsep.34.6.808 23204360

[B7] Browne R. A. Costa E. C. Sales M. M. Fonteles A. I. Moraes J. F. Barros Jde F. (2016). Acute effect of vigorous aerobic exercise on the inhibitory control in adolescents. *Rev. Paul. Pediatr.* 34 154–161. 10.1016/j.rpped.2015.08.004 26564328 PMC4917265

[B8] Chang Y. K. Chu C. H. Wang C. C. Wang Y. C. Song T. F. Tsai C. L. (2015). Dose-response relation between exercise duration and cognition. *Med. Sci. Sports Exerc.* 47 159–165. 10.1249/MSS.0000000000000383 24870572

[B9] Chang Y.-K. Etnier J. L. (2009). Exploring the dose-response relationship between resistance exercise intensity and cognitive function. *J. Sport Exerc. Psychol.* 31 640–656. 10.1123/jsep.31.5.640 20016113

[B10] Chang Y. K. Labban J. D. Gapin J. I. Etnier J. L. (2012). The effects of acute exercise on cognitive performance: a meta-analysis. *Brain Res.* 1453 87–101. 10.1016/j.brainres.2012.02.068 22480735

[B11] Chmura J. Nazar K. Kaciuba-Uscilko H. (1994). Choice reaction time during graded exercise in relation to blood lactate and plasma catecholamine thresholds. *Int. J. Sports Med.* 15 172–176. 10.1055/s-2007-1021042 8063464

[B12] Chu C.-H. Alderman B. L. Wei G.-X. Chang Y.-K. (2015). Effects of acute aerobic exercise on motor response inhibition: an ERP study using the stop-signal task. *J. Sport Health Sci.* 4 73–81. 10.1016/j.jshs.2014.12.002

[B13] Cooper S. B. Bandelow S. Nute M. L. Dring K. J. Stannard R. L. Morris J. G. (2016a). Sprint-based exercise and cognitive function in adolescents. *Prev. Med. Rep.* 4 155–161. 10.1016/j.pmedr.2016.06.004 27413677 PMC4929070

[B14] Cooper S. B. Dring K. J. Nevill M. E. (2016b). High-intensity intermittent exercise: effect on young people’s cardiometabolic health and cognition. *Curr. Sports Med. Rep.* 15 245–251. 10.1249/JSR.0000000000000273 27399821

[B15] Cragg L. Gilmore C. (2014). Skills underlying mathematics: the role of executive function in the development of mathematics proficiency. *Trends Neurosci. Educ.* 3 63–68. 10.1016/j.tine.2013.12.001

[B16] Curlik D. M. Shors T. J. (2013). Training your brain: Do mental and physical (MAP) training enhance cognition through the process of neurogenesis in the hippocampus? *Neuropharmacology* 64 506–514. 10.1016/j.neuropharm.2012.07.027 22898496 PMC3445739

[B17] Davranche K. Brisswalter J. Radel R. (2015). Where are the limits of the effects of exercise intensity on cognitive control? *J. Sport Health Sci.* 4 56–63. 10.1016/j.jshs.2014.08.004

[B18] Diamond A. (2013). Executive functions. *Annu. Rev. Psychol.* 64 135–168. 10.1146/annurev-psych-113011-143750 23020641 PMC4084861

[B19] Diamond A. (2015). Effects of physical exercise on executive functions: going beyond simply moving to moving with thought. *Ann. Sports Med. Res.* 2:1011. PMC4437637 26000340

[B20] Domazet S. L. Tarp J. Huang T. Gejl A. K. Andersen L. B. Froberg K. (2016). Associations of physical activity, sports participation and active commuting on mathematic performance and inhibitory control in adolescents. *PLoS ONE* 11:e0146319 10.1371/journal.pone.0146319 PMC4699746 26727211

[B21] Dorsey K. Herrin J. Krumholz H. Irwin M. (2009). The utility of shorter epochs in direct motion monitoring. *Res. Q. Exerc. Sport* 80 460–468. 10.1080/02701367.2009.10599584 19791632 PMC3152374

[B22] Ferris L. T. Williams J. S. Shen C. L. (2007). The effect of acute exercise on serum brain-derived neurotrophic factor levels and cognitive function. *Med. Sci. Sports Exerc.* 39 728–734. 10.1249/mss.0b013e31802f04c7 17414812

[B23] Golden C. J. (1994). *STROOP: Test de Colores y Palabras: Manual*. Madrid: TEA ediciones.

[B24] Griffin E. W. Mullally S. Foley C. Warmington S. A. O’Mara S. M. Kelly A. M. (2011). Aerobic exercise improves hippocampal function and increases BDNF in the serum of young adult males. *Physiol. Behav.* 104 934–941. 10.1016/j.physbeh.2011.06.005 21722657

[B25] Harveson A. T. Hannon J. C. Brusseau T. A. Podlog L. Papadopoulos C. Durrant L. H. (2016). Acute effects of 30 minutes resistance and aerobic exercise on cognition in a high school sample. *Res. Q. Exerc. Sport* 87 214–220. 10.1080/02701367.2016.1146943 26958898

[B26] Heyman E. Gamelin F.-X. Goekint M. Piscitelli F. Roelands B. Leclair E. (2012). Intense exercise increases circulating endocannabinoid and BDNF levels in humans—possible implications for reward and depression. *Psychoneuroendocrinology* 37 844–851. 10.1016/j.psyneuen.2011.09.017 22029953

[B27] Howie E. K. Schatz J. Pate R. R. (2015). Acute effects of classroom exercise breaks on executive function and math performance: a dose-response study. *Res. Q. Exerc. Sport* 86 217–224. 10.1080/02701367.2015.1039892 26009945

[B28] Koutsandreou F. Wegner M. Niemann C. Budde H. (2016). Effects of motor versus cardiovascular exercise training on children’s working memory. *Med. Sci. Sports Exerc.* 48 1144–1152. 10.1249/MSS.0000000000000869 26765631

[B29] Kremer M. M. Reichert F. F. Hallal P. C. (2012). Intensity and duration of physical efforts in physical education classes. *Rev. Saude Publica* 46 320–326. 10.1590/S0034-89102012005000014 22331179

[B30] Lee T. M. Wong M. L. Lau B. W. Lee J. C. Yau S. Y. So K. F. (2014). Aerobic exercise interacts with neurotrophic factors to predict cognitive functioning in adolescents. *Psychoneuroendocrinology* 39 214–224. 10.1016/j.psyneuen.2013.09.019 24149089

[B31] Liu-Ambrose T. Nagamatsu L. S. Graf P. Beattie B. L. Ashe M. C. Handy T. C. (2010). Resistance training and executive functions: a 12-month randomized controlled trial. *Arch. Intern. Med.* 170 170–178. 10.1001/archinternmed.2009.494 20101012 PMC3448565

[B32] Llorens F. Sanabria D. Huertas F. (2015). The influence of acute intense exercise on exogenous spatial attention depends on physical fitness level. *Exp. Psychol.* 62 20–29. 10.1027/1618-3169/a000270 25270559

[B33] McMorris T. Hale B. J. (2015). Is there an acute exercise-induced physiological/biochemical threshold which triggers increased speed of cognitive functioning? A meta-analytic investigation. *J. Sport Health Sci* 4 4–13. 10.1016/j.jshs.2014.08.003

[B34] Niemann C. Wegner M. Voelcker-Rehage C. Holzweg M. Arafat A. M. Budde H. (2013). Influence of acute and chronic physical activity on cognitive performance and saliva testosterone in preadolescent school children. *Ment. Health Phys. Act* 6 197–204. 10.1016/j.mhpa.2013.08.002

[B35] Pachana N. A. Thompson L. W. Marcopulos B. A. Yoash-Gantz R. (2004). California older adult Sroop test (COAST). Development of a stroop test adapted for geriatric populations. *Clin Gerontol* 27 3–22. 10.1300/J018v27n03_02

[B36] Phillips C. Baktir M. A. Srivatsan M. Salehi A. (2014). Neuroprotective effects of physical activity on the brain: a closer look at trophic factor signaling. *Front. Cell Neurosci.* 8:170 10.3389/fncel.2014.00170 PMC4064707 24999318

[B37] Piepmeier A. T. Shih C.-H. Whedon M. Williams L. M. Davis M. E. Henning D. A. (2015). The effect of acute exercise on cognitive performance in children with and without ADHD. *J. Sport Health Sci.* 4 97–104. 10.1016/j.jshs.2014.11.004

[B38] Pontifex M. B. Parks A. C. Henning D. A. Kamijo K. (2015). Single bouts of exercise selectively sustain attentional processes. *Psychophysiology* 52 618–625. 10.1111/psyp.12395 25523887 PMC4398582

[B39] Rodríguez Barreto L. C. Pineda Roa C. A. Pulido N. D. C. (2016). Propiedades psicométricas del Stroop, test de colores y palabras en población colombiana no patológica. *Univ. Psychol.* 15 255–272. 10.11144/Javeriana.upsy15-2.ppst

[B40] Sallis J. F. McKenzie T. L. Kolody B. Lewis M. Marshall S. Rosengard P. (1999). Effects of health-related physical education on academic achievement: project SPARK. *Res. Q. Exerc. Sport* 70 127–134. 10.1080/02701367.1999.10608030 10380244

[B41] Sandroff B. M. Hillman C. H. Benedict R. H. Motl R. W. (2016). Acute effects of varying intensities of treadmill walking exercise on inhibitory control in persons with multiple sclerosis: a pilot investigation. *Physiol. Behav.* 154 20–27. 10.1016/j.physbeh.2015.11.008 26569451

[B42] Santos-Lozano A. Santin-Medeiros F. Cardon G. Torres-Luque G. Bailon R. Bergmeir C. (2013). Actigraph GT3X: validation and determination of physical activity intensity cut points. *Int. J. Sports Med.* 34 975–982. 10.1055/s-0033-1337945 23700330

[B43] Schmit C. Davranche K. Easthope C. S. Colson S. S. Brisswalter J. Radel R. (2015). Pushing to the limits: the dynamics of cognitive control during exhausting exercise. *Neuropsychologia* 68 71–81. 10.1016/j.neuropsychologia.2015.01.006 25576908

[B44] Schmolesky M. T. Webb D. L. Hansen R. A. (2013). The effects of aerobic exercise intensity and duration on levels of brain-derived neurotrophic factor in healthy men. *J. Sports Sci. Med.* 12 502–511. 24149158 PMC3772595

[B45] Sibley B. A. Etnier J. L. Le Masurier G. C. (2006). Effects of an acute bout of exercise on cognitive aspects of Stroop performance. *J. Sport Exerc. Psychol.* 28 285–299. 10.1123/jsep.28.3.285

[B46] Soga K. Shishido T. Nagatomi R. (2015). Executive function during and after acute moderate aerobic exercise in adolescents. *Psychol. Sport Exerc.* 16 7–17. 10.1016/j.psychsport.2014.08.010

[B47] Stuss D. T. (1992). Biological and psychological development of executive functions. *Brain Cogn.* 20 8–23. 10.1016/0278-2626(92)90059-U 1389124

[B48] Sweat V. Yates K. F. Migliaccio R. Convit A. (2017). Obese adolescents show reduced cognitive processing speed compared with healthy weight peers. *Child Obes.* 13 190–196. 10.1089/chi.2016.0255 28256922 PMC5444419

[B49] Szuhany K. L. Bugatti M. Otto M. W. (2015). A meta-analytic review of the effects of exercise on brain-derived neurotrophic factor. *J. Psychiatr. Res.* 60 56–64. 10.1016/j.jpsychires.2014.10.003 25455510 PMC4314337

[B50] Thomas J. R. Nelson J. K. Silverman S. J. (eds). (2015). “Chapter 4. Formulatin the method,” in *Research Methods in Physical Activity* 7th Edn (Champaign, IL: Human Kinetics).

[B51] Torres-Luque G. Fernandez I. L. Santos-Lozano A. Garatachea N. Carnero E. Á. (2014). Actividad física y acelerometría; orientaciones metodológicas, recomendaciones y patrones. *Nutr. Hosp.* 31 115–128. 25561104 10.3305/nh.2015.31.1.7450

[B52] Treuth M. S. Schmitz K. Catellier D. J. McMurray R. G. Murray D. M. Almeida M. J. (2004). Defining accelerometer thresholds for activity intensities in adolescent girls. *Med. Sci. Sports Exerc.* 36 1259–1266. 15235335 PMC2423321

[B53] Troiano R. P. Berrigan D. Dodd K. W. Masse L. C. Tilert T. McDowell M. (2008). Physical activity in the United States measured by accelerometer. *Med. Sci. Sports Exerc.* 40 181–188. 10.1249/mss.0b013e31815a51b3 18091006

[B54] Tsai C. L. Chen F. C. Pan C. Y. Wang C. H. Huang T. H. Chen T. C. (2014). Impact of acute aerobic exercise and cardiorespiratory fitness on visuospatial attention performance and serum BDNF levels. *Psychoneuroendocrinology* 41 121–131. 10.1016/j.psyneuen.2013.12.014 24495613

[B55] Tsukamoto H. Suga T. Takenaka S. Tanaka D. Takeuchi T. Hamaoka T. (2016). Greater impact of acute high-intensity interval exercise on post-exercise executive function compared to moderate-intensity continuous exercise. *Physiol. Behav.* 155 224–230. 10.1016/j.physbeh.2015.12.021 26723268

[B56] van Uffelen J. G. Chinapaw M. J. van Mechelen W. Hopman-Rock M. (2008). Walking or vitamin B for cognition in older adults with mild cognitive impairment? A randomized controlled trial. *Br. J. Sports Med.* 42 344–351. 10.1136/bjsm.2007.044735 18308888

[B57] Verburgh L. Konigs M. Scherder E. J. Oosterlaan J. (2014). Physical exercise and executive functions in preadolescent children, adolescents and young adults: a meta-analysis. *Br. J. Sports Med.* 48 973–979. 10.1136/bjsports-2012-091441 23467962

[B58] Weinberg L. Hasni A. Shinohara M. Duarte A. (2014). A single bout of resistance exercise can enhance episodic memory performance. *Acta Psychol.* 153 13–19. 10.1016/j.actpsy.2014.06.011 PMC4597774 25262058

[B59] Weng T. B. Pierce G. L. Darling W. G. Voss M. W. (2015). Differential effects of acute exercise on distinct aspects of executive function. *Med. Sci. Sports Exerc.* 47 1460–1469. 10.1249/MSS.0000000000000542 25304335

[B60] Winter B. Breitenstein C. Mooren F. C. Voelker K. Fobker M. Lechtermann A. (2007). High impact running improves learning. *Neurobiol. Learn. Mem.* 87 597–609. 10.1016/j.nlm.2006.11.003 17185007

